# Endometrial compaction is associated with the outcome of artificial frozen-thawed embryo transfer cycles: a retrospective cohort study

**DOI:** 10.1007/s10815-023-02809-9

**Published:** 2023-05-04

**Authors:** Wenhan Ju, Chunxiao Wei, Xiaoliu Lu, Shuai Zhao, Jingyan Song, Hao Wang, Yi Yu, Shan Xiang, Fang Lian

**Affiliations:** 1 Shandong University of Traditional Chinese Medicine, Jinan, 250000 Shandong China; 2 Affiliated Hospital of Shandong University of Traditional Chinese Medicine, Jinan, 250011 Shandong China

**Keywords:** Endometrial compaction, Endometrial thickness, Frozen-thawed embryo transfer, Clinical pregnancy rate, Endometrial receptivity

## Abstract

**Introduction:**

The relationships between the outcome of frozen-thaw embryo transfer (FET) cycle and endometrial compaction were not quite consistent.

**Objective:**

To analyze the relationship between the outcome of FET cycle and endometrial compaction.

**Materials and methods:**

A total of 1420 women using FET were researched. The change in endometrial thickness on ET day and those on the day of progesterone (P) administration start is the basis for grouping. Group 1 was endometrial compaction group, and group 2 was the endometrial non-compaction group. Outcome measure was clinical pregnancy, estradiol (E_2_) levels, progesterone (P) levels, endometrial morphology, and thickness in each period of FET cycle.

**Results:**

A significantly lower clinical pregnancy rate was observed in group 2 in comparison with group 1 (43.4% vs. 55.1%, *P* < 0.01). In addition, P levels on the day of P administration start were lower in group 2 (0.73 ± 0.93 ng/ml vs. 0.90 ± 1.85 ng/ml, *P* = 0.006), while E_2_ levels on ET day were higher in group 2 (316.42 ± 304.95 pg/ml vs. 257.88 ± 219.15 pg/ml, *P* = 0.001) than in group 1. The binary logistic regression analysis showed a lower rate of clinical pregnancy in group 2 (aOR = 0.617, 95% CI 0.488-0.779, *P* = 0.001).

**Conclusions:**

Clinical pregnancy rates were significantly higher in women with endometrial compaction on ET day compared to women with no changes or thickening. Therefore, we recommend paying closer attention to endometrial compaction in women undergoing FET as a method to estimate endometrial receptivity.

## Introduction

In vitro fertilization-embryo transfer (IVF-ET) technology is widely used to treat infertility worldwide, and improving clinical pregnancy rates remains a hot research topic in the field of reproduction. After four decades of research, the rate of high-quality embryos in IVF-ET has increased to 70% [1]. In addition to embryo quality, endometrial tolerance is another key factor affecting the pregnancy rate. Low endometrial tolerance and altered embryo-endometrial dialog are believed to account for most transfer failures [2–4].

During infertility treatment, clinicians often assess the endometrium by transvaginal ultrasound and transabdominal ultrasound to evaluate endometrial receptivity. Assessing endometrial receptivity by ultrasound involves measuring various parameters, including endometrial thickness (EMT) [5], endometrial volume [6], endometrial pattern [7], and endometrial wave-like activity [8]. Most previous studies have focused on EMT and have revealed an association between an EMT of <7 mm and poor pregnancy outcomes [9–11].

Endometrial compaction, first proposed by Haas, Zilberberg et al. in 2018, refers to the decrease in EMT on the day of embryo transfer (ET) compared to the starting day of progesterone (P) administration in frozen-thawed embryo transfer (FET) cycles. Endometrial compaction could be associated with progesterone and an increased rate of sustained pregnancy after endometrial compaction [12]. Since then, seven other studies have focused on endometrial compaction [13–19]. However, the results were inconsistent, with four studies showing that endometrial compaction predicted good pregnancy outcomes [13, 15, 16, 18], and 3 studies showing no correlation with pregnancy outcomes [14, 17, 19]. However, these studies involved a small number of cases (<300 cases), which may have a major impact on the results. This large sample study aimed to comprehensively assess the relationship between endometrial compaction and pregnancy outcomes in women undergoing FET.

## Materials and methods

### Patient population and study design

This study was approved by the Ethics Committee of Reproductive Medicine of the Affiliated Hospital of Shandong University of Traditional Chinese Medicine, and informed consent was obtained from all the participants. The clinical data of FET patients attending the Department of Reproduction and Genetics from January 1, 2020 to June 30, 2022 were collected for a retrospective cohort study.

The eligibility criteria included the following: (1) age 20-49 years and (2) artificial cycle. The exclusion criteria were as follows: (1) other concurrent systemic diseases such as Cushing’s syndrome and pituitary tumors; (2) chromosomal abnormalities in either partner; (3) endometriosis; (4) congenital uterine malformations or untreated endometrial lesion; (5) donor cycles; and (6) pre-implantation genetic testing (PGT).

### Ovarian stimulation and oocyte extraction

Ovarian stimulation protocols were selected based on a combination of factors, including basal follicle-stimulating hormone (FSH) levels, patient age, BMI, and sinus follicle count. The treatments mainly included the GnRH antagonist regimen or the GnRH agonist regimen. For patients with reduced ovarian reserve, a mild ovarian stimulation protocol was used. As the target follicle diameter reached 18 mm or more, oocytes were extracted 34-36 h after induction with injectable chorionic gonadotropin (HCG, Zhuhai Livzon) and/or triptorelin acetate injection (Diphereline®, Ferring). Routine IVF or ICSI was performed 4-6 h later, according to the parameters of the male semen. The quality of embryos was graded by a professional embryo culturist; embryos at the cleavage stage were graded according to the Istanbul Consensus [20], and blastocysts were graded according to Gardner’s evaluation methods [21]. We regard embryos of cleavage stage I and blastocyst level 3BB and above as high-quality embryos.

### Endometrial preparation and assessment protocol

All patients were seen on day 3 of their menstrual cycle for serum reproductive hormone measurements and vaginal ultrasound, and oral estradiol valerate tablets (Progynova, Germany BAYER) 4-8 mg/day were started after the physician was informed of the hormonal and basal endometrial status. After 9 days of medication, the EMT and endometrial pattern were monitored daily by vaginal ultrasound, and serum E_2_ and P levels were measured. Intramuscular progesterone injection (China ZHEJIANG XIANJU) of 40 mg/day were administered after the endometrium reached a thickness of 7 mm or more. Patients who failed to meet the standard of progesterone administration continued to take oral estradiol valerate tablets, and those who did not reach 7 mm before endometrial transformation during the whole cycle were excluded from this study. The first day of progesterone injection was recorded as P+0, and the EMT on P+0 was recorded as T1. According to the patient’s decision, one to two cleavage embryos were transferred 3 days later (P+3), or one blastocyst was transferred 5 days later (P+5). The EMT evaluated by transabdominal ultrasonography on ET day was recorded as T2. Subsequently, progesterone injection (China ZHEJIANG XIANJU) and oral dydrogesterone tablets (Duphaston®, Netherlands Abbott Biologicals B.V.) were given for luteal support on that day. To minimize inaccuracies, EMT and morphology were measured by the same experienced ultrasonographer at our center for the entire FET cycle using a Voluson E10 device (GE Healthcare, Australia). As reported in a previous study [12], the criterion for endometrial compaction (*EC*) was set as 5%. Participants meeting this criterion were classified into group 1, while the others were classified into group 2. The formula for endometrial compaction is as follows.

$$\mathrm{EC}=\frac{-\left(\mathrm T2-\mathrm T1\right)}{\mathrm T1}\times100\%$$

### Outcome indicators

The primary outcome was clinical pregnancy. Secondary outcomes included the EMT, endometrial pattern, E_2_ and P levels on the first day of P administration and ET day, and the number of days of estrogen administration prior to progesterone addition. The EMT was measured in the median sagittal section of the uterus, and the largest EMT was measured on the bilateral anterior and posterior sides. Endometrial morphology was classified into A, B, and C according to the Gonen typing criteria [22], and participants with unclear ultrasound images were excluded. Transvaginal ultrasound was performed from 28 to 35 days after ET. Clinical pregnancy was defined as the presence of a gestational sac in the uterus detected by ultrasound with a germinal bud and primordial heartbeat within the sac.

### Statistical analysis

SPSS 25.0 was used for data analysis. In addition, group 1 was further subgrouped into 1A, 1B, and 1C with EC cut-off values of 5%, 10%, and 15%, respectively. P+3 and P+5 ET cycles were divided into 4 subgroups according to whether the endometrium was compacted or not: 3A (P+3 compaction), 3B (P+3 non-compaction), 5A (P+5 compaction), and 5B (P+5 non-compaction) for subgroup analysis. The measurement data were expressed in the form of mean ± SD (*x̅* ± *s*), and the independent sample *t*-test was conducted for comparisons between two groups, while a one-way ANOVA was used for comparisons between three groups. Categorical data were expressed as frequencies and percentages, and the chi-square test and Fisher exact test were performed for comparison. Post hoc pairwise comparisons were performed by Bonferroni correction. Binary logistic regression analysis was used to assess the effect of endometrial compaction on clinical pregnancy outcomes. Based on recently published clinical studies [23–25], age, body mass index (BMI), anti-Mullerian hormone (AMH), the number of quality embryos transferred, and the total number of embryos transferred were included in the analysis as potential confounders. Group 1 (endometrial compaction) was selected as the reference group. In the study, *P* < 0.05 was considered statistically significant.

## Results

### Baseline characteristics

**Fig. 1:**
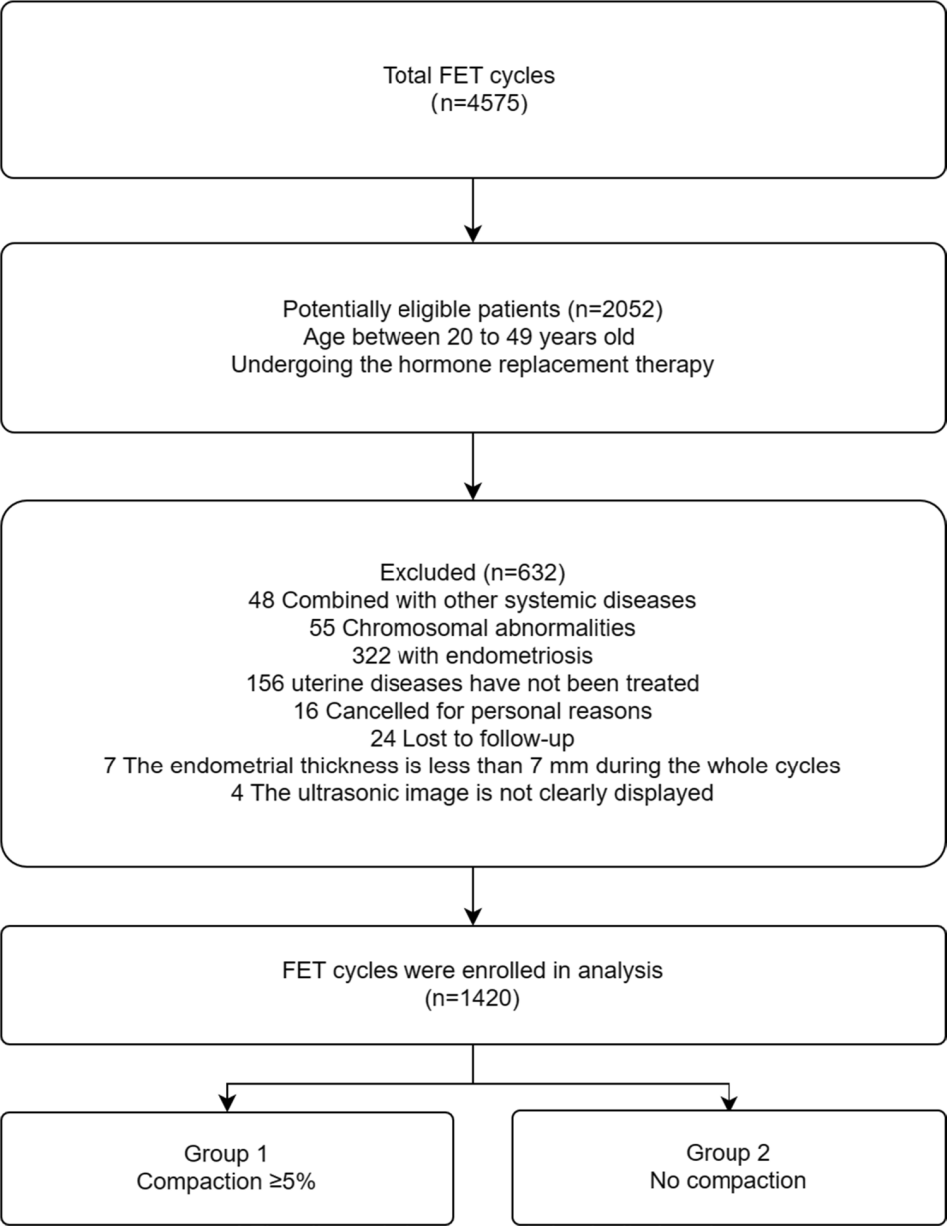
Flowchart of this study

**Fig. 2:**
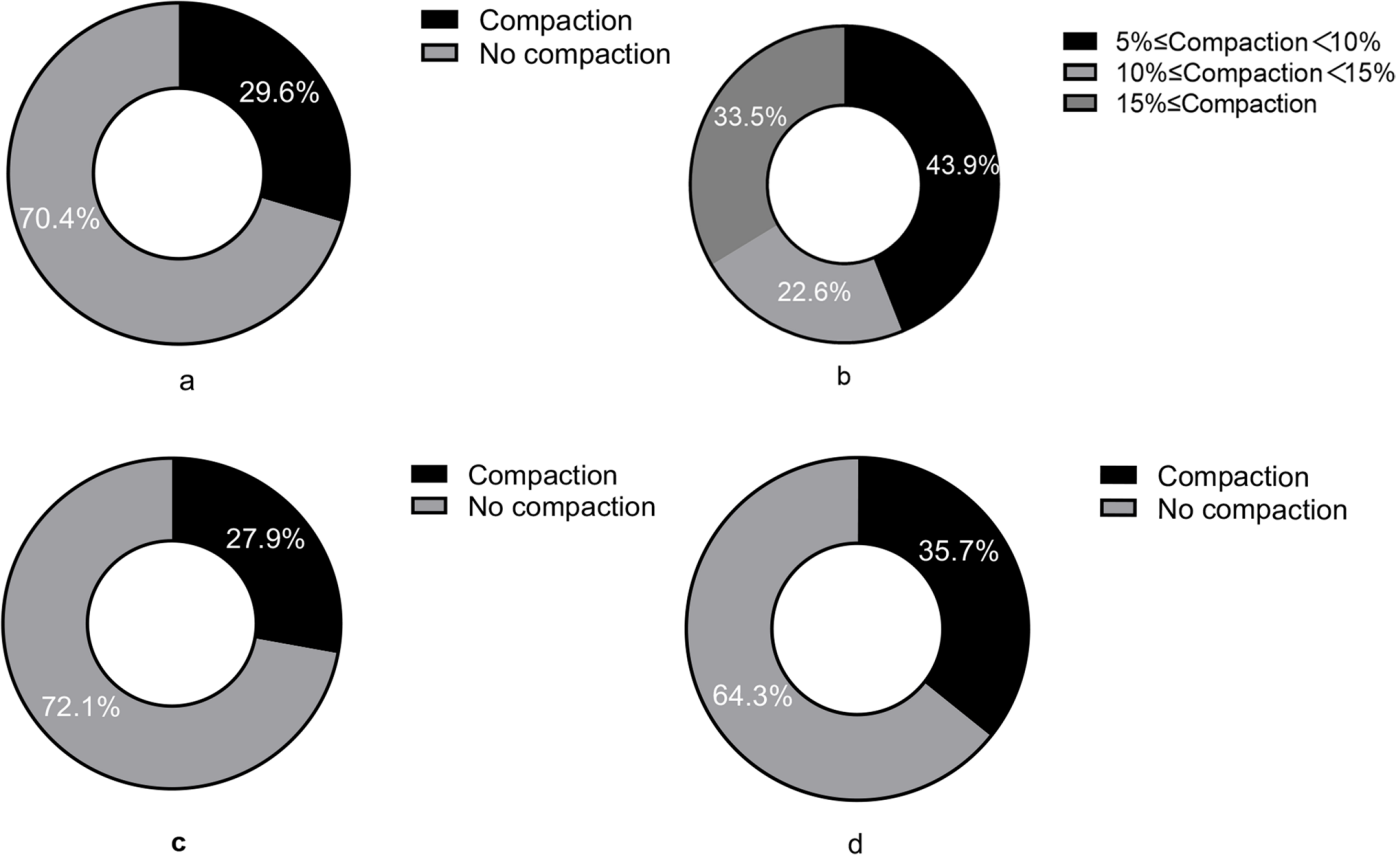
Endometrial compaction ratio

Only 1420 cycles met the study criteria, as displayed in Fig. 1. Of the 1420 cycles, 421 (29.6%) patients showed EMT compaction, and 999 (70.4%) patients showed essentially unchanged or increased EMT, as shown in Fig. 2a.Of the 421 cases, 185 (43.9%) had endometrial compaction ≥5% and <10%; 95 (22.6%) had endometrial compaction ≥10% and <15%; and 141 (33.5%) had endometrial compaction ≥15%, as shown in Fig. 2b. Of the P+3 ET cycles, 306 (27.9%) patients have endometrial compaction (Fig. 2c), and of P+5 ET cycles ,115 (35.7%) patients have endometrial compaction (Fig. 2d). No statistically significant differences (*P* > 0.05) were found in the baseline data in terms of age, type of infertility, years of infertility, infertility factors, BMI, basal endocrine characteristics, the total number of embryos transferred, and the number of quality embryos transferred between groups 1 and 2 (Table 1), and subgroups 1A, 1B, and 1C (Table 2). The number of high-quality embryos transferred in subgroups 5A and 5B was significantly lower than that in groups 3A and 3B. There was no statistical difference in other baseline data, as shown in Table 3.

**Table 1:** Comparison of basic characteristics between the two groups

	Group1	Group2	*P*
*n*	421	999	
Age (y)	32.45 ± 4.45	32.43 ± 4.29	0.343
Type of infertility			
Primary infertility (%)	43.9% (185/421)	40.0% (400/999)	0.172
Secondary infertility (%)	56.1% (236/421)	60.0% (599/999)	0.172
Years of infertility(y)	3.63 ± 2.61	3.47 ± 2.42	0.120
Infertility factors			
Fallopian tube factor (%)	98.6% (415/421)	98.8% (987/999)	0.730
Male factor (%)	9.0% (38/421)	9.9% (99/999)	0.606
Immunological factors (%)	0.2% (1/421)	0.1% (1/999)	0.528
Unspecified reasons (%)	0.5% (2/421)	0.1% (1/999)	0.160
Repeated implant failure (%)	6.7% (28/421)	7.3% (73/999)	0.660
Recurrent spontaneous abortion (%)	0.00	0.6% (6/999)	0.188
BMI (kg/m^2^)	24.15 ± 3.80	24.00 ± 3.66	0.183
AMH(ng/ml)	5.33 ± 3.20	5.36 ± 3.15	0.064
FSH (mIU/ml)	6.91 ± 2.94	7.09 ± 2.79	0.844
LH (mIU/ml)	6.37 ± 4.09	6.59 ± 4.58	0.690
E_2_ (pg/ml)	36.92 ± 14.55	37.95 ± 12.67	0.182
P (ng/ml)	0.38 ± 0.18	0.35 ± 0.18	0.656
T (ng/ml)	0.49 ± 0.35	0.48 ± 0.30	0.577
Basal endometrial thickness (mm)	5.37 ± 1.33	5.33 ± 1.21	0.130
Number of embryos transferred	1.66 ± 0.47	1.68 ± 0.47	0.139
Number of quality embryos transferred	0.58 ± 0.61	0.54 ± 0.60	0.806
Previous embryo transfer times	0.68 ± 1.07	0.79 ± 1.20	0.285

**Table 2:** Comparison of the basic characteristics of the three subgroups

	Group 1A	Group 1B	Group 1C	*P*
*n*	185	95	141	
Age (y)	32.55 ± 4.62	31.99 ± 4.42	32.62 ± 4.26	0.516
Type of infertility				
Primary infertility (%)	46.5% (86/185)	47.4% (45/95)	38.3% (54/141)	0.251
Secondary infertility (%)	53.5% (99/185)	52.6% (50/95)	61.7% (87/141)	0.251
Years of infertility(y)	3.67 ± 2.69	3.61 ± 2.38	3.60 ± 2.67	0.969
Infertility factors				
Fallopian tube factor (%)	98.9% (183/185)	100.0% (95/95)	97.2% (137/141)	0.171
Male factor (%)	9.2% (17/185)	6.3% (6/95)	10.6% (15/141)	0.521
Immunological factors (%)	0.00	0.00	0.7% (1/141)	0.561
Unspecified reasons (%)	0.00	0.00	1.4% (2/141)	0.162
Repeated implant failure (%)	7.6% (14/185)	7.4% (7/95)	5.0% (7/141)	0.614
BMI (kg/m^2^)	24.34 ± 3.73	23.49 ± 3.80	24.33 ± 3.87	0.158
AMH (ng/ml)	5.19 ± 3.05	5.10 ± 3.12	5.68 ± 3.42	0.281
FSH (mIU/ml)	6.87 ± 2.53	7.08 ± 2.77	6.85 ± 3.51	0.821
LH (mIU/ml)	6.36 ± 4.34	6.71 ± 3.78	6.17 ± 3.96	0.608
E_2_ (pg/ml)	37.02 ± 13.67	38.47 ± 17.72	35.76 ± 13.25	0.374
P (ng/ml)	0.37 ± 0.18	0.40 ± 0.17	0.39 ± 0.18	0.316
T (ng/ml)	0.48 ± 0.40	0.51 ± 0.30	0.49 ± 0.31	0.727
Basal endometrial thickness (mm)	5.28 ± 1.17	5.32 ± 1.50	5.52 ± 1.38	0.248
Number of embryos transferred	1.65 ± 0.48	1.66 ± 0.48	1.66 ± 0.47	0.928
Number of quality embryos transferred	0.62 ± 0.59	0.59 ± 0.61	0.55 ± 0.62	0.511
Previous embryo transfer times	0.63 ± 0.96	0.60 ± 1.12	0.76 ± 1.12	0.384

**Table 3:** Comparison of the basic characteristics of the four subgroups

	3A	3B	5A	5B	*P*
*n*	306	792	115	207	
Age (y)	32.55 ± 4.45	32.45 ± 4.26	32.19 ± 4.47	32.35 ± 4.38	0.884
Type of infertility					
Primary infertility (%)	45.1% (138/306)	41.5% (329/792)	40.9% (47/115)	34.3% (71/207)	0.110
Secondary infertility (%)	54.9% (168/306)	58.5% (463/792)	59.1% (68/115)	65.7% (136/207)	0.110
Years of infertility (y)	3.71 ± 2.66	3.55 ± 2.40	3.44 ± 2.48	3.19 ± 2.47	0.130
Infertility factors					
Fallopian tube factor (%)	98.4% (301/306)	98.5% (780/792)	99.1% (114/115)	100.0% (207/207)	0.272
Male factor (%)	9.5% (29/306)	11.1% (88/792)	7.8% (9/115)	5.3% (11/207)	0.070
Immunological factors (%)	0	0.1% (1/792)	0.9% (1/115)	0	0.286
Unspecified reasons (%)	0.3% (1/306)	0.1% (1/792)	0.9% (1/115)	0	0.231
Repeated implant failure (%)	6.2% (19/306)	7.7% (61/792)	7.8% (9/115)	5.8% (12/207)	0.700
Recurrent spontaneous abortion (%)	0	0.5% (4/792)	0	1.0% (2/207)	0.363
BMI (kg/m^2^)	24.22 ± 3.79	24.08 ± 3.68	23.95 ± 3.84	23.70 ± 3.56	0.466
AMH (ng/ml)	5.21 ± 3.21	5.28 ± 3.01	5.65 ± 3.15	5.67 ± 3.64	0.253
FSH (mIU/ml)	6.90 ± 3.21	7.10 ± 2.42	6.94 ± 2.05	7.04 ± 3.90	0.750
LH (mIU/ml)	6.37 ± 4.33	6.51 ± 4.15	6.38 ± 3.38	6.88 ± 5.96	0.620
E_2_ (pg/ml)	37.10 ± 13.40	38.01 ± 12.69	36.45 ± 17.28	37.72 ± 12.58	0.560
P (ng/ml)	0.38 ± 0.18	0.36 ± 0.18	0.38 ± 0.18	0.35 ± 0.18	0.083
T (ng/ml)	0.49 ± 0.36	0.49 ± 0.31	0.48 ± 0.32	0.46 ± 0.28	0.693
Basal endometrial thickness (mm)	5.28 ± 1.21	5.31 ± 1.20	5.60 ± 1.57	5.37 ± 1.25	0.111
Number of quality embryos transferred	0.64 ± 0.63	0.58 ± 0.62	0.43 ± 0.50	0.39 ± 0.49	0.001
Previous embryo transfer times	0.68 ± 1.01	0.76 ± 1.13	0.68 ± 1.22	0.92 ± 1.42	0.125

### Cycle characteristics

Table 4 depicts the characteristics of FET cycles in groups 1 and 2. The progesterone levels (0.73 ± 0.93 ng/ml vs. 0.90 ± 1.85 ng/ml, *P* = 0.006), B-endometrial pattern ratio (20.1% vs. 25.2%, *P* = 0.034), and C-endometrial pattern ratio (0.4% vs. 1.9%, *P* = 0.005) on the first day of P administration in group 2 were significantly lower than in group 1. Furthermore, the rate of pattern A endometrium on the first day of P administration (79.5% vs. 72.9%, *P* = 0.007), E_2_ levels on ET day (316.42 ± 304.95 pg/ml vs. 257.88 ± 219.15 pg/ml, *P* = 0.001), and EMT on ET day (10.17 ± 1.67 mm vs. 9.11 ± 1.48 mm, *P* = 0.016) were significantly higher than in group 1.

**Table 4:** Comparison of cycle characteristics between the two groups

	Group 1	Group 2	*P*
Number	421	999	
E_2_ on the day of P administration start (pg/ml)	266.83 ± 364.02	295.34 ± 283.83	0.767
P on the day of P administration start (ng/ml)	0.90 ± 1.85	0.73 ± 0.93	0.006
EMT on the day of P administration start (mm)	10.52 ± 1.71	9.55 ± 1.62	0.185
Endometrial pattern on the day of P administration start			
A endometrium on the day of P administration start (%)	72.9% (307/421)	79.5% (794/999)	0.007
B endometrium on the day of P administration start (%)	25.2% (106/421)	20.1% (201/999)	0.034
C endometrium on the day of P administration start (%)	1.9% (8/421)	0.4% (4/999)	0.005
E_2_ on embryo transfer day (pg/ml)	257.88 ± 219.15	316.42 ± 304.95	0.001
P on embryo transfer day (ng/ml)	10.66 ± 6.05	10.54 ± 6.27	0.976
EMT on embryo transfer day (mm)	9.11 ± 1.48	10.17 ± 1.67	0.016
Endometrial pattern			
A endometrium on embryo transfer day (%)	1.4% (6/421)	0.7% (7/999)	0.190
B endometrium on embryo transfer day (%)	27.8% (117/421)	29.0% (290/999)	0.637
C endometrium on embryo transfer day (%)	70.8% (298/421)	70.3% (702/999)	0.846
Days of estrogen administration before P	11.93 ± 3.34	11.85 ± 3.32	0.644

Table 5 describes the characteristics of FET cycles in groups 1A, 1B, and 1C. The E_2_ levels on the first day of P administration in group 1C (205.37 ± 202.44 pg/ml) were significantly lower than those in group 1A (292.61 ± 365.65 pg/ml) and group 1B (307.85 ± 510.54 pg/ml) (*P* < 0.05). Moreover, the EMT on the first day of P administration was significantly higher in group 1C (11.11 ± 1.70 mm) than in group 1A (10.12 ± 1.57 mm) and group 1B (10.45 ± 1.77 mm). The EMT on the first day of P administration and on ET day was significantly lower in group 1C (8.61 ± 1.34 mm) compared to group 1A (9.44 ± 1.47 mm) and group 1B (9.21 ± 1.54 mm).

**Table 5:** Comparison of cycle characteristics among the three subgroups

	Group 1A	Group 1B	Group 1C	*P*
Number	185	95	141	
E_2_ on the day of P administration start (pg/ml)	292.61 ± 365.65*	307.85 ± 510.54^*^	205.37 ± 202.44	0.046
P on the day of P administration start (ng/ml)	0.89 ± 2.42	0.90 ± 1.21	0.91 ± 1.23	0.998
EMT on the day of P administration start (mm)	10.12 ± 1.57**	10.45 ± 1.77^**^	11.11 ± 1.70	0.001
Endometrial pattern on the day of P administration start				
A endometrium on the day of P administration start (%)	75.1% (139/185)	72.6% (69/95)	70.2% (99/141)	0.610
B endometrium on the day of P administration start (%)	24.3% (45/185)	25.3% (42/95)	26.2% (37/141)	0.925
C endometrium on the day of P administration start (%)	0.5% (1/185)	2.1% (2/95)	3.5% (5/141)	0.110
E_2_ on embryo transfer day (pg/ml)	284.78 ± 219.74	245.72 ± 228.34	230.78 ± 209.31	0.073
P on embryo transfer day (ng/ml)	10.64 ± 6.20	10.99 ± 5.74	10.47 ± 6.09	0.811
EMT on embryo transfer day (mm)	9.44 ± 1.47^**^	9.21 ± 1.54^**^	8.61 ± 1.34	0.001
Endometrial pattern				
A endometrium on embryo transfer day (%)	1.6% (3/185)	1.1% (1/95)	1.4% (2/141)	0.999
B endometrium on embryo transfer day (%)	23.2% (43/185)	29.5% (28/95)	32.6% (46/141)	0.159
C endometrium on embryo transfer day (%)	75.1% (139/185)	69.5% (66/95)	66.0% (93/141)	0.186
Days of estrogen administration before P	11.98 ± 3.28	11.80 ± 3.52	11.94 ± 3.31	0.909

^*^Compared with group 1C, *P* < 0.05

^**^Compared with group 1C, *P* < 0.01

Table 6 describes the cycle characteristics of groups 3A, 3B, 5A, and 5B. Preliminary analysis shows that there may be differences between subgroups in the EMT on the day of P administration start, A endometrium on the first day of P administration, C endometrium on the first day of P administration, E_2_ on embryo transfer day, and EMT on embryo transfer day. Further comparison between 3A/3B and 5A/5B showed that E_2_ level and EMT embryo transfer day in 3B group were significantly lower than those in 3A group, while other data had no statistical difference (*P* > 0.05).

**Table 6:** Comparison of cycle characteristics among the three subgroups

	3A	3B	5A	5B	*P*
*n*	306	792	115	207	
E_2_ on day of start P administration (pg/ml)	258.38 ± 369.29	291.83 ± 275.06	289.30 ± 350.19	308.73 ± 315.50	0.281
P on day of start P administration (ng/ml)	0.94 ± 2.08	0.71 ± 0.84	0.79 ± 1.00	0.79 ± 1.23	0.067
Endometrial thickness on day of start P administration (mm)	10.60 ± 1.75	9.60 ± 1.65	10.31 ± 1.59	9.41 ± 1.52	0.001
Endometrial pattern on day of start P administration					
A endometrium on the day of P administration start (%)	74.8% (229/306)	80.2% (635/792)	67.8% (78/115)	76.8% (159/207)	0.013
B endometrium on the day of P administration start (%)	24.2% (74/306)	19.6% (155/792)	27.8% (32/115)	22.2% (46/207)	0.121
C endometrium on the day of P administration start (%)	1.0% (3/306)	0.3% (2/792)	4.3% (5/115)	1.0% (2/207)	0.001
E_2_ on embryo transfer day (pg/ml)	257.68 ± 209.40	322.34 ± 314.42^*^	258.43 ± 244.19	293.74 ± 265.10	0.002
P on embryo transfer day (ng/ml)	10.81 ± 5.92	10.60 ± 6.50	10.27 ± 6.40	10.27 ± 5.26	0.739
Endometrial thickness on embryo transfer day (mm)	9.19 ± 1.50	10.15 ± 1.68**	8.90 ± 1.43	10.28 ± 1.67	0.001
Endometrial pattern					
A endometrium on embryo transfer day (%)	1.3% (4/306)	0.8% (6/792)	1.7% (2/115)	0.5% (1/207)	0.378
B endometrium on embryo transfer day (%)	26.1% (80/306)	28.2% (223/792)	32.2% (37/115)	32.4% (67/207)	0.364
C endometrium on embryo transfer day (%)	72.5% (222/306)	71.1% (563/792)	66.1% (76/115)	67.1% (139/207)	0.396
Days of estrogen administration before P	11.97 ± 3.34	11.84 ± 3.30	11.80 ± 3.34	11.88 ± 3.40	0.940

^*^Compared with group 3A, *P* < 0.05

^**^Compared with group 3A, *P* < 0.01

## Comparison of pregnancy outcomes

**Fig. 3:**
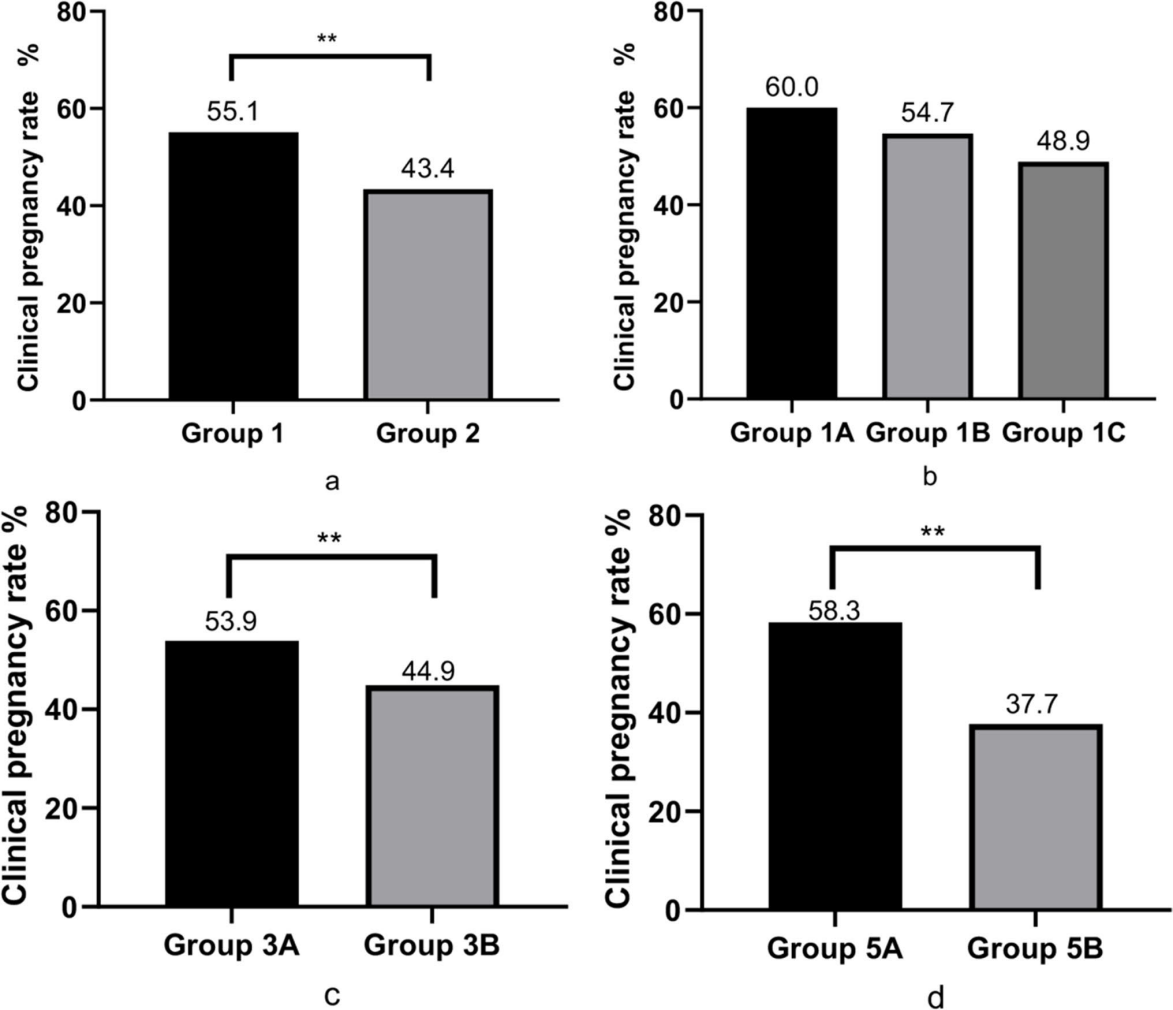
Comparison of clinical pregnancy rates

The clinical pregnancy rate in group 1 (55.1%) was significantly higher than that in group 2 (43.4%) (*P* < 0.01), as shown in Fig. 3a. In the subgroup analysis, no significant difference was found in the clinical pregnancy rate among the three groups, 1A, 1B, and 1C (*P* > 0.05), as illustrated in Fig. 3b. The clinical pregnancy rate of subgroup 3A (53.9%) was significantly higher than that of subgroup 3B (44.9%) (Fig. 3c), and the clinical pregnancy rate of subgroup 5A (58.3%) was significantly higher than that of subgroup 5B (37.7%) (Fig. 3d).

### Relationship between clinical pregnancy outcomes and endometrial compaction

The results of the binary logic regression analysis are shown in Table 7. A lower rate of clinical pregnancy was observed in group 2 compared with group 1 (adjusted odds ratio (aOR) = 0.617, 95% CI 0.488-0.779, *P* = 0.001), indicating that endometrial compaction was a positive predictor of clinical pregnancy. In addition, AMH (aOR = 1.066, 95% CI 1.026-1.107, *P* = 0.003), the number of transferred high-quality embryos (aOR = 1.278, 95% CI 1.062-1.539, *P* = 0.009), and the total number of transferred embryos (aOR = 1.355, 95% CI 1.066-1.722, *P* = 0.013) were positive predictors of clinical pregnancy, while age (aOR = 0.960, 95% CI 0.935-0.986, *P* = 0.003) was a risk factor for clinical pregnancy.

**Table 7:** Results of the binary logistic regression analysis

	aOR	95% confidence interval	*P*
Age	0.960	[0.935, 0.986]	0.003
BMI	0.999	[0.971, 1.029]	0.962
AMH	1.066	[1.026, 1.107]	0.001
Number of high-quality embryos transferred	1.278	[1.062, 1.539]	0.009
Total number of embryos transferred	1.355	[1.066, 1.722]	0.013
Groups			
Group 1	1.000 (reference)		
Group 2	0.617	[0.488, 0.779]	0.001

## Discussion

In this analysis of clinical data from 1420 FET cycles, clinical pregnancy rates were higher in women with endometrial compaction (≥5%) on the ET day compared to women with no change or endometrial thickening. Furthermore, the binary logistic analysis results also showed that endometrial compaction was a positive predictor of clinical pregnancy, suggesting good pregnancy outcomes. These findings are consistent with five previous studies [12, 13, 15, 16, 18], although three studies reported no correlation [14, 17, 19]. Moreover, our study seemed to show a trend towards a decrease in clinical pregnancy rate as the endometrial compaction rate (5%, 10%, 15%) increased, but the trend was not statistically significant. However, Zilberberg reported that the highest sustained pregnancy rate (51.5%) was observed at a 15% endometrial compaction rate [13], although this conclusion was not supported by other studies.

The endometrial functional layer undergoes changes to adapt to embryo implantation and is affected by estrogen and progesterone levels in the body, resulting in cyclic, regular shedding that leads to menstruation. During the proliferative phase, estrogen secretion increases to promote endometrial cell division and growth, which is reflected by increased EMT, glandular and vascular growth, and the typical three-layer appearance of A pattern endometrium on ultrasound. After ovulation, the corpus luteum is formed, which secretes progesterone in response to estrogen. In response to the hormonal changes, endometrial thickening is stopped, while glandular secretion and vascular tortuosity are increased, accompanied by glycogen accumulation [26] and NK cell proliferation [27]. At this point, the endometrium shows the C pattern, characterized by a homogeneous and strongly echogenic appearance on ultrasound, with an indistinguishable uterine cavity line. This change may explain endometrial compaction. In our study, the ET day E_2_ levels were significantly higher in group 2 (316.42 ± 304.95 pg/ml) than in group 1 (257.88 ± 219.15 pg/ml), P levels on the first day of P administration were significantly lower in group 2 (0.73 ± 0.93 ng/ml) compared to group 1 (0.90 ± 1.85 ng/ml), and the C-endometrial pattern ratio on the first day of P administration was significantly lower in group 2 (0.4%) compared to group 1 (1.9%). The results suggest that the negative effects of excessive estrogen and insufficient progesterone may affect endometrial compaction. Fluctuations in serum E2 and P levels may lead to a desynchronization between the embryo and endometrium, lowering the clinical pregnancy rate. A previous study reported that serum progesterone levels were not consistent with those in endometrial tissues [28, 29]. Our study demonstrated no significant difference in ET day progesterone levels between the two groups. Further speculated that progesterone resistance might also play a role in endometrial compaction. Current studies suggest that progesterone resistance is associated with chronic inflammation, progesterone receptor gene polymorphisms, and epigenetic alterations [30–32]. No statistically significant difference in progesterone level and endometrial type was found on ET day between the two groups. It is speculated that although the endometrial transformation was completed under progesterone action in both groups, estrogen predominantly affected the endometrium due to progesterone resistance, resulting in untimely endometrial compaction and altering the endometrial microenvironment, leading to a decrease in clinical pregnancy rate. Nevertheless, endometrial compaction with progestin resistance and changes in the endometrial microenvironment should be further explored to confirm this speculation.

In addition, our study found that P+5 ET cycles had a higher proportion of endometrial densification (35.7%) than P+3 ET cycles (27.9%). No matter P+3 or P+5 ET cycles, the clinical pregnancy rate of endometrial compaction group is higher than that of non-compaction group (*P* < 0.01). We found that the E_2_ on embryo transfer day in both 3A and 5A subgroups was relatively low, and the level of serum E_2_ was equivalent (257.68 ± 209.40 vs. 258.43 ± 244.19, *P* > 0.05). The E2 on embryo transfer day in 3B subgroup was higher than that in 5B subgroup (322.34 ± 314.42 vs. 293.74 ± 265.10, *P* > 0.05), and only showed statistical difference between 3A and 3B groups (257.68 ± 209.40 vs. 322.34 ± 314.42). There was no significant difference in progesterone levels among the four subgroups on the ET day (*P* > 0.05). This suggests that the prolongation of progesterone action time will lead to an increase in the proportion of endometrial compaction, but this effect may not be shown in the level of serum estradiol and progesterone on the day of transplantation, but will cause changes in the microenvironment of endometrium, leading to an increase in the rate of endometrial compaction.

Different methods of endometrial preparation, endometrial measurement, and the quality of transferred embryos may influence the outcome. Our study was consistent with 5 studies [12–14, 16, 19] that used artificial cycles for endometrial preparation, but the observations were not entirely consistent. Among them, the studies from Kaye et al. [16], Olgan et al. [19], and Riestenberg et al. [14] all used vaginal ultrasound to assess T2, but Riestenberg et al. [14] observed the EMT 1 day before ET. The rate of endometrial compaction on vaginal ultrasound ranged from 9.5 to 16.6%, whereas this rate ranged from 28.2 to 45% when T2 was measured by abdominal ultrasound, which is similar to the 29.6% measured in our study. Although vaginal ultrasound is more accurate in measuring EMT on ET day [19], measurement by abdominal ultrasound is more convenient and less invasive for transplantation. To minimize T2 measurement error, the same sonographer used the same type of machine for measurements throughout the FET cycle. In addition, most of the previous studies were performed during PGT cycles to observe the effect of endometrial compaction on FET outcomes [13, 14], aiming to reduce the effect of embryo quality on the outcome. However, multicenter clinical studies have shown that PGT does not improve live birth rates [33]. Embryo quality can be assessed morphologically and genetically as superior or inferior, and predicting its developmental potential is not perfect [34]. Our study did not take into account PGT cycles and considered the quantity and quality of transferred embryos while comparing basal characteristics, which is a more complete approach than previous studies.

The strengths of this study include the consideration of hormone level changes, the relationship between endometrial pattern and endometrial compaction, comparison of P+3 and P+5 ET cycles, and the larger sample size compared to previous studies. Nevertheless, the limitations of the study should be acknowledged. The use of abdominal ultrasound to measure T2 is less precise than vaginal ultrasound. Furthermore, the live birth rates, gestational disease rates, and offspring birth defect rates have been overlooked. Selection bias could be present due to the retrospective nature of the study. Therefore, a multicenter study with a more comprehensive and rigorous experimental design is required to confirm the findings in this study.

## Conclusion

In summary, clinical pregnancy rates were higher in women with endometrial compaction (≥5%) in FET cycles compared to women with no change or thickening. Failure of endometrial compaction is a risk factor for poor clinical pregnancy rates in FET cycles, presumably due to excessive serum estradiol levels and progesterone resistance. Therefore, we recommend paying more attention to endometrial compaction as a phenomenon in FET cycles to assess endometrial receptivity. In addition, long-term, multicenter, prospective studies with large samples are still needed to confirm our conclusions.

## Data Availability

The datasets analyzed during the current study are available from the corresponding author upon reasonable request.

## Abbreviations

*IVF/ICSI*: In vitro fertilization/intracytoplasmic sperm injection
*BMI*: Body mass index
*EMT*: Endometrial thickness
*AMH*: Anti-Mullerian hormone
*PGT*: Pre-implantation genetic testing
*FSH*: Follicle-stimulating hormone
*LH*: Luteinizing hormone
*E*_*2*_: Estradiol
*P*: Progesterone
*FET*: Frozen-thawed embryo transfer
*ET*: Embryo transfer
*MII*: Metaphase stage II
*SD*: Standard deviation
*aOR*: Adjusted odds ratios
*CI*: Confidence intervals

## References

[1] Sebastian-Leon P, Garrido N, Remohí J, Pellicer A, Diaz-Gimeno P. Asynchronous and pathological windows of implantation: two causes of recurrent implantation failure. Hum Reprod. 2018 Apr 1;33(4):626–35. 10.1093/humrep/dey023. 10.1093/humrep/dey023 29452422

[2] Edwards RG (1994). Implantation, interception and contraception. Hum Reprod..

[3] Simón C, Moreno C, Remohí J, Pellicer A (1998). Cytokines and embryo implantation. J Reprod Immunol..

[4] Franasiak JM, Forman EJ, Hong KH, Werner MD, Upham KM, Treff NR, Scott RT Jr. The nature of aneuploidy with increasing age of the female partner: a review of 15 169 consecutive trophectoderm biopsies evaluated with comprehensive chromosomal screening. Fertil Steril. 2014;101:656–63. 10.1016/j.fertnstert.2013.11.004. 10.1016/j.fertnstert.2013.11.004 24355045

[5] Gallos ID, Khairy M, Chu J, Rajkhowa M, Tobias A, Campbell A, Dowell K, Fishel S, Coomarasamy A. Optimal endometrial thickness to maximize live births and minimize pregnancy losses: analysis of 25,767 fresh embryo transfers. Reprod Biomed Online. 2018;37:542–8. 10.1016/j.rbmo.2018.08.025. 10.1016/j.rbmo.2018.08.025 30366837

[6] Zhang CH, Chen C, Wang JR, Wang Y, Wen SX, Cao YP (2022). An endometrial receptivity scoring system basing on the endometrial thickness, volume, echo, peristalsis, and blood flow evaluated by ultrasonography. Front Endocrinol (Lausanne)..

[7] Yang W, Zhang T, Li Z, Ren X, Huang B, Zhu G (2018). Combined analysis of endometrial thickness and pattern in predicting clinical outcomes of frozen embryo transfer cycles with morphological good-quality blastocyst: a retrospective cohort study. Medicine (Baltimore)..

[8] Saito R, Kajihara T, Takamura M, Tochigi H, Sato T, Ishihara O (2020). High stretch cycling inhibits the morphological and biological decidual process in human endometrial stromal cells. Reprod Med Biol..

[9] Liao Z, Liu C, Cai L, Shen L, Sui C, Zhang H (2022). The effect of endometrial thickness on pregnancy, maternal, and perinatal outcomes of women in fresh cycles after IVF/ICSI: a systematic review and meta-analysis. Front Endocrinol (Lausanne)..

[10] Zhang T, Li Z, Ren X, Huang B, Zhu G, Yang W (2018). Endometrial thickness as a predictor of the reproductive outcomes in fresh and frozen embryo transfer cycles: a retrospective cohort study of 1512 IVF cycles with morphologically good-quality blastocyst. Medicine (Baltimore)..

[11] von Wolff M, Fäh M, Roumet M, Mitter V, Stute P, Griesinger G (2018). Thin endometrium is also associated with lower clinical pregnancy rate in unstimulated menstrual cycles: a study based on natural cycle IVF. Front Endocrinol (Lausanne)..

[12] Haas J, Smith R, Zilberberg E, Nayot D, Meriano J, Barzilay E (2019). Endometrial compaction (decreased thickness) in response to progesterone results in optimal pregnancy outcome in frozen-thawed embryo transfers. Fertil Steril..

[13] Zilberberg E, Smith R, Nayot D, Haas J, Meriano J, Barzilay E (2020). Endometrial compaction before frozen euploid embryo transfer improves ongoing pregnancy rates. Fertil Steril..

[14] Riestenberg C, Quinn M, Akopians A, Danzer H, Surrey M, Ghadir S (2021). Endometrial compaction does not predict live birth rate in single euploid frozen embryo transfer cycles. J Assist Reprod Genet..

[15] Jin Z, Li J, Yang E, Shi H, Bu Z, Niu W (2021). Effect of endometrial thickness changes on clinical pregnancy rates after progesterone administration in a single frozen-thawed euploid blastocyst transfer cycle using natural cycles with luteal support for PGT-SR- and PGT-M-assisted reproduction: a retrospective cohort study. Reprod Biol Endocrinol..

[16] Kaye L, Rasouli MA, Liu A, Raman A, Bedient C, Garner FC (2021). The change in endometrial thickness following progesterone exposure correlates with in vitro fertilization outcome after transfer of vitrified-warmed blastocysts. J Assist Reprod Genet..

[17] Shah JS, Vaughan DA, Dodge LE, Leung A, Korkidakis A, Sakkas D (2022). Endometrial compaction does not predict live birth in single euploid frozen embryo transfers: a prospective study. Hum Reprod..

[18] Yaprak E, Şükür YE, Özmen B, Sönmezer M, Berker B, Atabekoğlu C (2021). Endometrial compaction is associated with the increased live birth rate in artificial frozen-thawed embryo transfer cycles. Hum Fertil (Camb)..

[19] Olgan S, Dirican EK, Sakinci M, Caglar M, Ozsipahi AC, Gul SM (2022). Endometrial compaction does not predict the reproductive outcome after vitrified-warmed embryo transfer: a prospective cohort study. Reprod Biomed Online..

[20] Alpha Scientists in Reproductive Medicine and ESHRE Special Interest Group of Embryology (2011). The Istanbul consensus workshop on embryo assessment: proceedings of an expert meeting. Hum Reprod..

[21] Gardner DK, Lane M, Stevens J, Schlenker T, Schoolcraft WB (2000). Blastocyst score affects implantation and pregnancy outcome: towards a single blastocyst transfer. Fertil Steril..

[22] Gonen Y, Casper RF (1990). Prediction of implantation by the sonographic appearance of the endometrium during controlled ovarian stimulation for in vitro fertilization (IVF). J In Vitro Fert Embryo Transf..

[23] Li D, Hu Z, Chen Q, Chai W, Cai R, Kuang Y (2022). Neonatal outcomes and congenital malformations in children born after progestin-primed ovarian stimulation protocol. Front Endocrinol (Lausanne)..

[24] Talbot AL, Alexopoulou E, Kallemose T, Freiesleben NC, Nielsen HS, Zedeler A (2022). Binucleated embryos at the two-cell stage show higher blastocyst formation rates and higher pregnancy and live birth rates compared to non-multinucleated embryos. Hum Reprod Open..

[25] Volodarsky-Perel A, Ton Nu TN, Orvieto R, Mashiach R, Machado-Gedeon A, Cui Y (2022). The impact of embryo vitrification on placental histopathology features and perinatal outcome in singleton live births. Hum Reprod..

[26] Mizutani S, Matsumoto K, Kato Y, Mizutani E, Mizutani H, Iwase A (2020). New insights into human endometrial aminopeptidases in both implantation and menstruation. Biochim Biophys Acta Proteins Proteom..

[27] Radović Janošević D, Trandafilović M, Krtinić D, Čolović H, Milošević Stevanović J, Pop-Trajković DS (2020). Endometrial immunocompetent cells in proliferative and secretory phase of normal menstrual cycle. Folia Morphol (Warsz)..

[28] Usadi RS, Groll JM, Lessey BA, Lininger RA, Zaino RJ, Fritz MA (2008). Endometrial development and function in experimentally induced luteal phase deficiency. J Clin Endocrinol Metab..

[29] Lawrenz B, Fatemi HM (2022). Are serum progesterone measurements truly representative for the identification of an adequate luteal phase in hormonal replacement therapy frozen embryo transfers?. Hum Reprod..

[30] MacLean JA, Hayashi K (2022). Progesterone actions and resistance in gynecological disorders. Cells..

[31] Patel BG, Rudnicki M, Yu J, Shu Y, Taylor RN (2017). Progesterone resistance in endometriosis: origins, consequences and interventions. Acta Obstet Gynecol Scand..

[32] Pu H, Wen X, Luo D, Guo Z (2022). Regulation of progesterone receptor expression in endometriosis, endometrial cancer, and breast cancer by estrogen, polymorphisms, transcription factors, epigenetic alterations, and ubiquitin-proteasome system. J Steroid Biochem Mol Biol..

[33] Yan J, Qin Y, Zhao H, Sun Y, Gong F, Li R (2021). Live birth with or without preimplantation genetic testing for aneuploidy. N Engl J Med..

[34] Van Soom A, Mateusen B, Leroy J, De Kruif A (2003). Assessment of mammalian embryo quality: what can we learn from embryo morphology?. Reprod Biomed Online..

